# Peripheral Blood Cells and Clinical Profiles as Biomarkers for Pain Detection in Palliative Care Patients

**DOI:** 10.3390/biomedicines14010176

**Published:** 2026-01-14

**Authors:** Hugo Ribeiro, Raquel Alves, Joana Jorge, Bárbara Oliveiros, Tânia Gaspar, Inês Rodrigues, João Rocha Neves, Joana Brandão Silva, António Pereira Neves, Ana Bela Sarmento-Ribeiro, Marília Dourado, Ana Cristina Gonçalves, José Paulo Andrade

**Affiliations:** 1 Community Palliative Care Support Team Gaia, Health Local Unit Gaia and Espinho, 4434-502 Vila Nova de Gaia, Portugal; 2 Faculty of Medicine (FMUC), University of Coimbra, 3004-504 Coimbra, Portugal; 3 Group of Environment, Genetics and Oncobiology (CIMAGO)—FMUC, Center for Innovative Biomedicine and Biotechnology (CIBB), Coimbra Institute for Clinical and Biomedical Research (iCBR), 3000-548 Coimbra, Portugal; 4 Departamento de Medicina da Comunidade, Informação e Decisão em Saúde, Faculty of Medicine, University of Porto, 4200-393 Porto, Portugal; 5 Centro Académico Clínico, 3004-504 Coimbra, Portugal; 6 Unit of Anatomy, Department of Biomedicine, Faculty of Medicine, University of Porto, 4200-393 Porto, Portugal; 7 RISE-Health, 4200-319 Porto, Portugal; 8 School of Medicine and Biomedical Sciences, University of Porto, 4200-393 Porto, Portugal; 9 Vascular Service, Local Health Unit S. João, 4200-319 Porto, Portugal; 10 Clinical Hematology Department, Local Health Unit of Coimbra, 3004-561 Coimbra, Portugal

**Keywords:** monocytes, platelets, biomarkers, pain assessment, dementia, renal insufficiency, aged, malnutrition

## Abstract

**Background/Objectives:** Patients in need of specialized palliative care are clinically highly complex, with pain being the most prevalent problem. Furthermore, in these patients, a self-report for characterization of pain could be difficult to obtain. This cross-sectional, exploratory study investigates the use of clinical parameters and peripheral blood biomarkers for potentially identifying and characterizing pain (assessed using Pain Assessment in Advanced Dementia (PAINAD) and Numeric Scale (NS)) in patients under palliative care, including a population with dementia where pain is often underdiagnosed. **Methods:** Fifty-three patients with non-oncological diseases were analyzed in a cross-sectional study using medical and nursing records. Among previous biomarkers related to monocytes and platelets assessed by flow cytometry, we selected the most significative ones for pain characterization in a logistic regression analysis (multivariate analysis), alongside patient-specific characteristics such as renal function, nutritional status, and age. **Results:** Our exploratory findings suggest strong relationships between chronic pain and advanced age, reduced glomerular filtration rate (GFR), and malnutrition within this cohort. Furthermore, the percentage of lymphocytes, total and classical monocytes, the relative expression in monocytes of CD206, CD163, the CD163/CD206 ratio, and the relative expression in platelets of CD59 emerged as potential predictors of pain. Statistical analyses highlighted the challenges of multicollinearity among variables such as age, GFR, and nutritional status. A classification model further suggested that all patients over 65 years in our specific sample reported pain. **Conclusions:** This pilot study provides preliminary support for prior evidence linking chronic pain to aging, nutritional deficits, and renal impairment, and highlights potential novel peripheral blood biomarkers for pain assessment. This work emphasizes the promise of clinical and molecular biomarkers to improve pain detection and management, contributing to personalized and effective palliative care strategies.

## 1. Introduction

Regardless of pathologies and comorbidities, patients in need of specialized palliative care are patients with high clinical complexity [1], with pain being the most prevalent problem [2,3]. Home-specialized palliative care is usually provided for patients with severe–extreme frailty who do not benefit from hospital follow-ups or benefit from being monitored by other hospital specialties, given the trajectory of the disease [4,5].

In patients with dementia in advanced stages, reliably identifying and characterizing pain is a significant challenge. Self-reporting, a cornerstone of pain assessment, is seriously compromised in these individuals [6], making it difficult to ascertain pain location, intensity, associated symptoms, impact on daily life, and response to established therapies. Numerical scales (NS), while valuable for cognitively intact patients, rely entirely on subjective report and cannot be used effectively in those with communication difficulties or cognitive impairment [7]. While observational scales, such as the Pain Assessment in Advanced Dementia Scale (PAINAD) [7,8], can be employed, and their observational nature means they assess behavioral indicators rather than the subjective pain experience itself. Consequently, PAINAD often provides only a probability of the presence or absence of pain, lacking the specificity to characterize pain type, intensity nuances, or precise location, and may not capture all forms of pain [8]. This inherent limitation of traditional assessment methods frequently leads to the underdiagnosis and undertreatment of pain in this vulnerable population, highlighting a critical unmet clinical need for more objective and reliable assessment tools. However, characterizing pain is essential for a targeted, effective, and safe pharmacological approach [9,10].

Biomarkers are measurable biological characteristics that help with prediction, diagnosis, and prognosis, and treat diseases by identifying targets for developing new drugs [11,12]. The discovery of biomarkers that help to identify, evaluate, characterize, and monitor pain is of utmost relevancy [13,14]. However, despite multiple efforts, it has not yet been possible to identify robust biomarkers of pain, while on the other hand, there are studies underway with imaging [15,16,17], genomic and epigenomic [18,19,20], biological [17,21] and electrophysiological biomarkers [22,23]. While promising candidates have emerged in various pain conditions, the specific context of palliative care presents unique complexities [1,2]. Patients in palliative care often experience multi-morbidities, frailty, and cognitive impairments—such as advanced dementia—that profoundly impact their ability to communicate pain. Consequently, biomarkers effective in general pain populations may not be directly applicable, or sufficiently sensitive and specific, for accurate pain detection and management in this vulnerable and heterogeneous group [1,2,3]. This highlights a critical unmet need for objective biological indicators of pain tailored to the palliative care setting.

Platelets have a proven fundamental role in hemostasis, thrombosis, healing, and cancer. Furthermore, its important role in the inflammatory and immune reaction has been advanced [24,25]. The potential role of platelets in nociception and pain is already recognized, as their granules store and release substances such as serotonin, histamine and bradykinin [26,27]. Some platelet membrane receptors, such as cluster of differentiation (CD) 62p (P-selectin) and CD41 (glycoprotein (GP) IIb-IIIa) have been identified as markers of platelet activation [28]. Beyond these established markers, we also investigated others reflecting specific platelet functions relevant to inflammation and immune regulation. For instance, CD36 (Glycoprotein IV), a scavenger receptor, is implicated in inflammatory responses and platelet adhesion. CD40, a member of the TNF receptor superfamily, is expressed on activated platelets and contributes to immune cell activation and inflammatory signaling. CD59, an inhibitor of the membrane attack complex, is crucial for protecting cells from complement-mediated lysis, and its expression on platelets can be modulated during inflammatory processes or oxidative stress, potentially influencing platelet function in chronic pain states [24,25,29].

Monocytes are cells involved in the promotion and resolution of inflammatory processes [30]. Three subtypes of monocytes have been described, namely classical (approximately 85%), non-classical (approximately 10%), and intermediate (approximately 5%), which have differences in CD14 and CD16 expression [31,32]. These subtypes have different roles in homeostasis, inflammation, and various pain-causing diseases, such as rheumatoid arthritis, complex regional pain syndrome, malignant neoplasms, acquired immunodeficiency syndrome (AIDS), and tuberculosis [31,33,34,35]. Monocytes present several molecules, receptors, and cell signaling pathways that can be used as pain markers. Some studies have demonstrated a significant increase in intermediate monocytes in inflammatory diseases [31,32]. Upon differentiation into macrophages or during activation, these cells can adopt distinct functional phenotypes, broadly categorized as M1 (pro-inflammatory) and M2 (anti-inflammatory/pro-resolving) states. M1 monocytes/macrophages are typically activated by factors like lipopolysaccharide and IFN-γ, leading to the production of pro-inflammatory cytokines (e.g., TNF-α, IL-6, IL-1β) that contribute to tissue damage and the initiation of acute pain. Conversely, M2 monocytes/macrophages, often induced by IL-4 or IL-13, are associated with the resolution of inflammation, tissue repair, immune regulation, and the production of anti-inflammatory mediators (e.g., IL-10). A sustained M1-dominant response or a failure in M2-mediated resolution can perpetuate chronic inflammation and contribute significantly to the chronification of pain [31,36,37,38]. Specifically, CD163 and CD206 are scavenger receptors highly expressed on distinct monocyte/macrophage populations, particularly those associated with an M2-like phenotype involved in inflammation resolution, tissue repair, and immunosuppression. Their modulated expression is implicated in various chronic inflammatory conditions and pain pathophysiology. Furthermore, CD11c and CD86 are co-stimulatory molecules whose expression varies with monocyte activation states, reflecting their capacity to initiate or modulate immune responses relevant to nociception [30,31,32,39,40,41,42].

In our previous studies, we have identified separately, in a univariate analysis, pain biomarkers in platelets [29] and in monocytes and its subsets [39]. However, which pain peripheral blood cell and molecular biomarker is most relevant and the correlation with clinical parameters have not been characterized.

This study aimed to identify, using a multivariate analysis, the best peripheral blood biomarkers among those evaluated previously in monocytes and platelets (specifically including CD36, CD40, CD59, CD11c, CD86, CD163, and CD206, selected based on their known roles in inflammation, immune modulation, and platelet/monocyte function in pain pathophysiology) to improve the characterization of pain in palliative patients with advanced dementia in the context of care provided by a community palliative support team. As secondary objectives, we examined how individual patient characteristics, including organ functional status (renal and liver function), body composition (weight and body mass index), and personal history, could aid in the early identification of pain.

## 2. Methods

Study design: Observational, analytic, transversal, non-interventional study using medical and nursing records of chronic pain patients.

Participants’ inclusion criteria and data collection: For the purpose of this study, chronic pain was operationally defined as pain persisting or recurring for more than 3 months, or pain lasting beyond the expected healing time of an acute injury, consistent with International Association for the Study of Pain (IASP) guidelines. The presence and characteristics of pain were ascertained through a comprehensive review of medical and nursing records. For patients capable of self-reporting, pain intensity was assessed using a Numeric Scale (NS) [43]. For patients with advanced dementia, where self-report was compromised, pain was identified and characterized using the Pain Assessment in Advanced Dementia (PAINAD) scale [7,8]. Only patients with documented active and continuous pain within the evaluation period were included in the ‘pain’ group for analysis. All patients with non-oncological diseases under clinical follow-up from a palliative care specialized team in the North of Portugal were selected. We initially selected 95 patients. However, the legal representatives of five patients refused to participate in this study. Seventeen patients were excluded from blood sample collection due to their fragile condition, hypovolemia, and difficult venous access. Also, 20 patients were in end-of-life, and we decided to avoid blood collection in this clinical condition. Individual and clinical data were reutilized from the clinical process of 53 palliative patients with non-oncological diseases and followed by a specialized palliative care team between 1 September and 31 December 2021. Blood samples were collected during morning visits (between 8:00 AM and 11:00 AM) to minimize diurnal variations. While strict fasting was not universally enforced due to patient comfort and the palliative care setting, collection times were standardized relative to patient routines. The residual blood samples were utilized for additional analyses. The data was registered in a protected Microsoft Excel^®^ sheet, and an alphanumeric code was used to identify each patient, thus keeping the identity confidential since only the researcher knows it. Following the European General Data Protection Regulation (GDPR), these electronic files will be deleted after the study’s end and the publication of the results.

Ethical approval: The Ethics Committees of the Faculty of Medicine of the University of Porto and the North Regional Health Administration of Portugal approved the research procedures, and the study was conducted following the Declaration of Helsinki. Before enrollment, participants or their legal representatives provided informed consent for participation. The international ethical guidelines of confidentiality, the anonymity of personal data, and the abandonment option were followed.

Variables under study: The primary outcome for this study was the binary presence or absence of chronic pain. For this purpose, data from both the Numeric Scale (NS) [43] and the Pain Assessment in Advanced Dementia (PAINAD) [8] were consolidated. Pain presence on NS was defined as a score > 0. For PAINAD, pain presence was defined as a score ≥ 5 (out of 10), indicating probable pain requiring intervention. Severity levels for PAINAD were categorized as <5 (no/mild pain), 5–7 (moderate pain), and 8–10 (severe pain). This combined approach was chosen to create a comprehensive binary outcome variable applicable across our heterogeneous patient population, facilitating biomarker discovery for pain detection irrespective of cognitive status.

Age, gender, type of pain (neuropathic, nociceptive, nociplastic, or mixed), analgesic drugs (opioid and other analgesics, such as nonsteroidal anti-inflammatory drugs (NSAIDs) and acetaminophen) and doses in use, type of dementia (Alzheimer, Vascular, Frontotemporal and Lewy body), glomerular filtration rate (GFR), nutritional status (using body mass index [BMI] and Mini Nutritional Assessment Scale [MNA] [44]), and functional and disease status (using Barthel scale [45] and palliative performance scale [PPS] [46]) were assessed. If the patient had the pain under control, it was registered for how long it was controlled.

In order to perform a multivariate analysis to identify the best cellular and/or molecular peripheral blood biomarker for pain characterization, among other patient clinical parameters, we selected the most significative results obtained in univariate analysis previously described by us [29,39] for the further analysis. These parameters were selected among those evaluated in perypheral blood by flow cytometry, namely in freshly prepared platelets [the glycoprotein IV (CD36), integrin α6 (CD49f), integrin β3 (CD61), P-selectin (CD62p), the complement activation inhibitor protein (CD59) and TNFα family receptor CD40] and in monocytes and its subsets, classical (CD14+/CD16-), non-classical (CD14-/CD16+) and intermediate (CD14+/CD16+) the transmembrane proteins CD11c, CD86, CD163, and CD206. For monocytes and lymphocytes, data are presented as percentages of the total leukocyte population or specific subsets, and for transmembrane proteins, data are presented as relative expression levels.

Flow cytometry analysis: Peripheral blood samples were processed for flow cytometry using standard protocols. Cells were initially identified based on Forward Scatter (FSC) and Side Scatter (SSC) characteristics to gate on the total leukocyte population, excluding debris and aggregates. Lymphocytes were then identified by their characteristic FSC/SSC profile. Monocytes were identified as a distinct population based on their larger size (higher FSC) and granularity (higher SSC) relative to lymphocytes, and this was further confirmed by their expression of specific markers such as CD14 and CD45. Monocyte subsets (classical, intermediate, non-classical) were further differentiated based on the differential expression of CD14 and CD16. Specific markers (CD206, CD163, CD59, CD36, CD40, CD11c, CD86) were analyzed using appropriate fluorescently conjugated antibodies and corresponding isotype controls within these phenotypically defined cell populations.

## 3. Statistical Analysis

The qualitative sample data were described using absolute and relative frequencies, while the quantitative values were described by the minimum and maximum together with the mean and standard deviation if they were normally distributed or by the median and quartiles in normality absence. Adjustment to the normal distribution was assessed using the Kurtosis and Skewness test.

The association between pain and qualitative variables was assessed using Fisher’s exact test, and the comparison of quantitative variables between patients with and without pain was carried out using Student’s t-test for independent samples when the distribution was normal in each of the groups (using the Welsh correction in the absence of heteroscedasticity assessed by the Levene test) or using the Mann–Whitney test in the absence of this assumption.

After identifying the variables with potential predictors of pain in this population, a logistic regression model was used to identify markers and biomarkers for pain after evaluating the multicollinearity between them by using the variance inflation factor (VIF) and analysis of the correlation and association between them using Pearson’s correlation coefficient in the case of two quantitative variables, Fisher’s exact test in the case of two qualitative ones, Student’s t-test with or without correction, and Welsh or Mann–Whitney if one of each type. Missing values were handled by list-wise deletion for specific analyses; thus, the sample size (n) for each reported biomarker result is explicitly specified when it deviates from the overall study population. Specifically, platelet CD59 was excluded from the multivariate analysis due to missing values (available for 46 out of 53 patients). This missingness (seven patients, approximately 13%) was largely systematic, attributed to challenges in sample quality and processing for this particular marker within our fragile palliative care cohort, making its robust inclusion in the multivariate model problematic.

Receiver operating characteristics (ROC) analysis was performed to identify cut-off points for these variables.

A tree format multivariable dependence analysis was conducted using a classification and regression tree algorithm to understand how some independent variables interacted with the dependent variable.

The analysis was carried out in Jamovi^®^, version 2.3, and was evaluated at a significance level of 5%.

## 4. Results

The sample consisted of 53 patients aged between 29 and 99 years, mostly of female gender (39 cases, 73.6%). According to PAINAD or NS, 44 patients (83.0%) felt pain, and among these, 38 had severe dementia.

Table 1 represents the characterization of patients’ samples without and with pain and the comparison between groups, according to gender, body mass index (BMI), glomerular filtration rate (GFR), nutritional state evaluated through the Mini Nutritional Assessment (MNA), and immune subsets and platelet indicators with statistical significance. Regarding the distribution in each of the groups, there is a balance in the distribution for gender (*p* = 0.092) and body mass index (BMI) (*p* = 0.334). However, the group experiencing pain tended to be older (*p* = 0.001), has a lower glomerular filtration rate (GFR) (*p* = 0.001), and exhibits poorer nutritional status (*p* = 0.001) compared to the pain-free group. The group with pain also exhibited the following:

○ Lower percentages of lymphocytes (21.29% vs. 29.06%, *p* = 0.042): This suggests a potential alteration in overall adaptive immune responses.
○ A complex shift in monocyte populations was observed, with higher total and classical monocyte percentages (5.71% vs. 4.19%, *p* = 0.045 for total; 91.6% vs. 85.27%, *p* = 0.03 for classical), but significantly lower percentages of intermediate and non-classical monocytes (3.14% vs. 5.71%, *p* = 0.008 for intermediate; 2.79% vs. 5.53%, *p* = 0.007 for non-classical). This pattern hints at a specific re-distribution or activation state within the monocyte compartment.
○ Markedly reduced expression of monocyte CD206 (8.01% vs. 14.08%, *p* = 0.017) and CD163 (87.99% vs. 95.63%, *p* = 0.011) (for total and classical monocytes, respectively), along with a lower CD163/CD206 ratio (4.79 vs. 9.50, *p* = 0.028): Given that these markers are often associated with M2-like, pro-resolving monocyte/macrophage phenotypes, these findings could imply a shift away from anti-inflammatory or resolution pathways in the presence of chronic pain.
○ The significantly lower expression of platelet CD59 (2.72% vs. 11.75%, *p* = 0.047) indicates potential alterations in platelet function or activation that might contribute to or reflect pain pathophysiology.

**Table 1:** Characterization of the patients’ samples without and with pain and comparison between groups.

Demographic and Clinical Characteristics	Without Pain (n = 9)	With Pain (n = 44)	*p*
Gender M/F	0/9 (0.0%/23.1%)	14/30 (100.0%/76.9%)	0.092 ^¥^
**Age, average (SD)**	**44.7 (12.03)**	**81.4 (10.80)**	**<0.001** ^§^
BMI kg/m^2^, average (SD)	25.2 (2.39)	24.2 (4.28)	0.334 ^⩯^
**GFR, average (SD)**	**115.0 (11.18)**	**57** **.4 (23.70)**	**<0.001** ^§^
**MNA, median (SD)**	**29.8 (0** **.67)**	**12** **.4 (7.4)**	**<0.001** ^⩩^
**Immune Subsets and Platelet Indicators**			
**Lymphocytes (% of leukocytes) Mean (±SD)**	**(n = 9)** **29.06 (±13.22)**	**(n = 39)** **21.29 (±9.24)**	**0.042**
**Monocytes (Total, % of leukocytes)**	**(n = 9)** **4.19 (±1.45)**	**(n = 39)** **5.71 (±2.09)**	**0.045**
**Classical Monocytes (% of monocytes)**	**(n = 9)** **85.27 (±5.39)**	**(n = 39)** **91.6 (±4.22)**	**0.03**
**Intermediate Monocytes (% of monocytes)**	**(n = 9)** **5.71 (±2.65)**	**(n = 39)** **3.14 (±1.98)**	**0.008**
**Non-Classical Monocytes (% of monocytes)**	**(n = 9)** **5.53 (±3.26)**	**(n = 39)** **2.79 (±2.50)**	**0.007**
**Monocyte CD206 (% expression)—total monocytes**	**(n = 9)** **14.08 (±7.80)**	**(n = 39)** **8.01 (±6.31)**	**0.017**
**Monocyte CD163 (% expression)—classical monocytes**	**(n = 9)** **95.63 (±6.26)**	**(n = 39)** **87.99 (±9.36)**	**0.011**
**Monocyte CD163/CD206 Ratio—total monocytes**	**(n = 9)** **9.50 (±5.69)**	**(n = 39)** **4.79 (±5.58)**	**0.028**
**Platelet CD59 (% expression)**	**(n = 5)** **11.75 (±7.20)**	**(n = 41)** **2.72 (±4.10)**	**0.047**

n = number; *p* = significance; M = male; F = female; SD = standard deviation; BMI = body mass index; GFR = glomerular filtration rate; MNA = Mini Nutritional Assessment; bold = characteristics with statistical significance; ^¥^ Fisher’s exact test; ^§^ Student’s t test for independent samples; ^⩯^ Student’s t-test for independent samples with correction and Welsh; ^⩩^ Mann–Whitney U test; the statistically significant results are highlighted in bold.

Collectively, these data suggest a distinct immune and inflammatory profile in palliative care patients with chronic pain, further reinforcing the potential of these peripheral blood cell parameters as novel, albeit exploratory, biomarkers for pain detection. This detailed presentation in Table 1 provides foundational quantitative data for these preliminary observations.

A comprehensive list of all measured immune and platelet biomarkers, including those not showing statistical significance, as well as details on medication usage, can be found in Supplementary Table S1.

Table 2 provides a descriptive characterization of pain (assessed by PAINAD or Numeric Scale (NS)) and other parameters relating to the degree of functionality (PPS) global performance (Barthel scale) and nutritional status (MNA), specifically within the 44 patients classified as having pain. It is important to note that scores from PAINAD and NS were analyzed separately within this descriptive table, reflecting the individual assessment methods used, and were not combined into a single, composite pain intensity score.

Besides the qualitative clinical variables described as potential predictors of pain, we also selected, for further analysis, the most quantitative predictive pain biomarkers among those obtained by flow cytometry previously described by us in platelets and monocytes in an univariate analysis [38,47], using a logistic regression model to identify possible biomarkers; a classification tree model was also developed to understand interactions between these biomarkers, as further explained.

We selected the most significative results (*p* < 0.05), namely the percentage of lymphocytes, total monocytes, classical monocytes, intermediate monocytes, non-classical monocytes, the relative expression in total monocytes of CD206 and CD163/CD206 ratio, the relative expression of CD163 in classic monocytes, and the relative expression of CD59 in platelets.

In patients with pain within our cohort, a statistically significant decrease was observed in the percentage of lymphocytes and intermediate and non-classic monocytes, alongside an increase in the percentage of total monocytes. Furthermore, a decrease in the percentage of total monocytes expressing CD206, in the ratio of CD163/CD206, and in CD163 expression in classical monocytes, was also noted in these patients.

After identifying the variables with potential predictors of pain in this population, as age, dementia, GFR, MNA, percentage of lymphocyte and total monocytes and its subsets [classical (CM), intermediate (IM), non-classical (NCM)] and monocytes markers (as the % of TM expressing CD206 and the %CD163/%CD206 ratio and of CD163 in CM), along with the percentage of platelets expressing CD59, a logistic regression model and a classification tree were constructed to identify predictors of pain in this population, after assessing multicollinearity between these using the variance inflation factor (VIF). However, as there are many missing values in platelets positive for CD59, this parameter was excluded from the multivariate analysis.

We identify a strong correlation between age, GFR, and MNA, as well as between the % TM expressing CD206 and the CD163/CD206 ratio, or between the percentage of lymphocytes and monocytes, which raises multicollinearity problems.

On the other hand, there are 12 variables with statistical significance (after excluding CD59 in platelets, for which only 46 samples were available), so including all of them would imply using a sample of at least 130 patients, ideally 195 cases, in order to have 10 to 15 cases for each independent variable.

**Figure 1:**
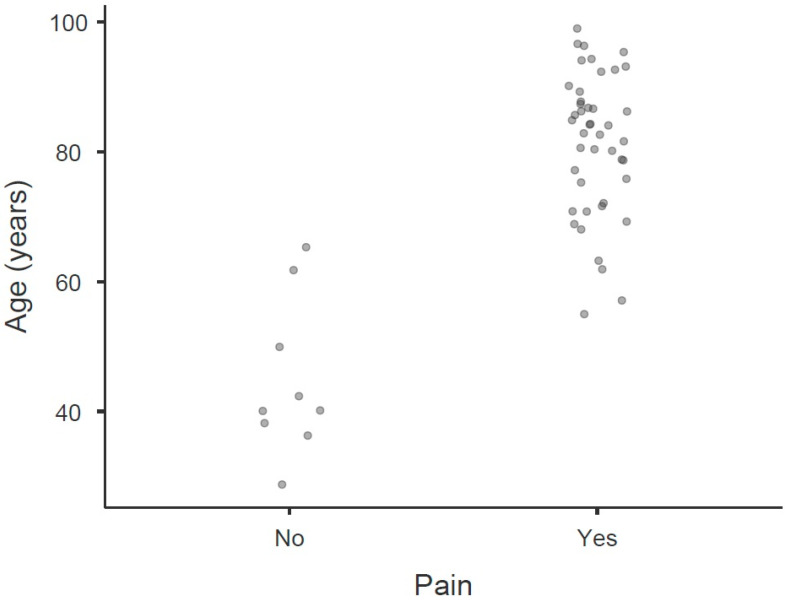

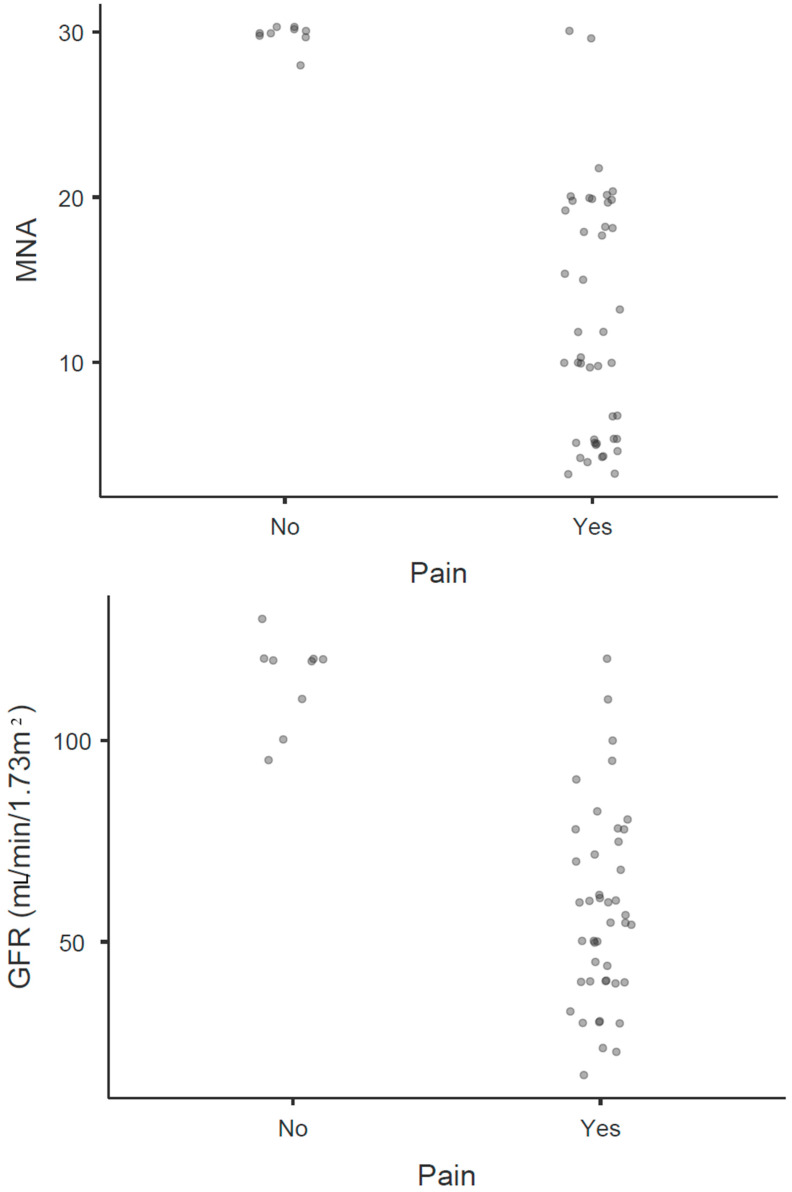
Main individual patient characteristics with strong relation with pain. This figure consists of three scatter dot plots that compare two groups, “Yes” and “No” responses related to pain, for three different variables: GFR (mL/min/1.73 m^2^), age (years), and MNA. Dot plot for GFR. The “No” group shows a higher concentration around higher values of GFR, whereas the “Yes” group spans a lower range of GFR values. Dot plot for age. The “No” group is concentrated around younger ages, while the “Yes” group’s ages are spread out more evenly. Dot plot for MNA. The “No” group is concentrated at higher MNA values, whereas the “Yes” group has a larger spread but trough lower values. GFR—glomerular filtration rate; MNA—mininutrional assessment score (values vary from 0 to 30).

**Figure 2:**
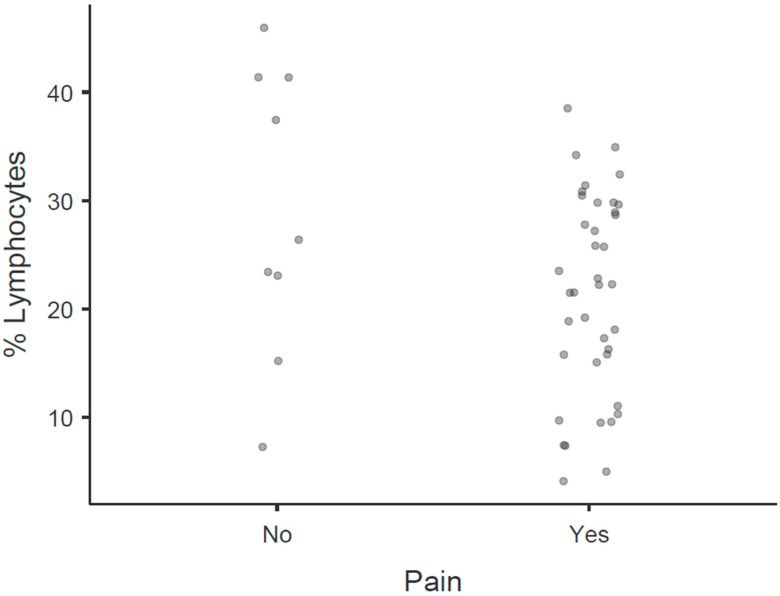

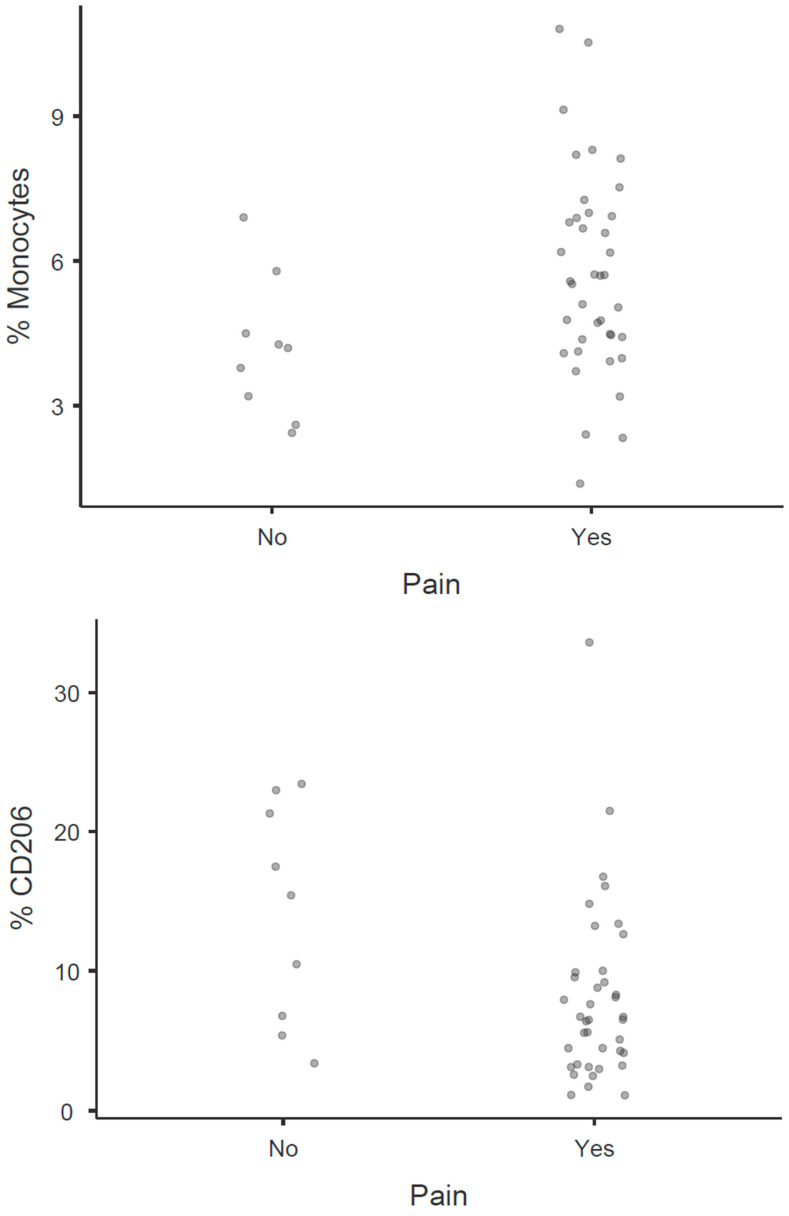

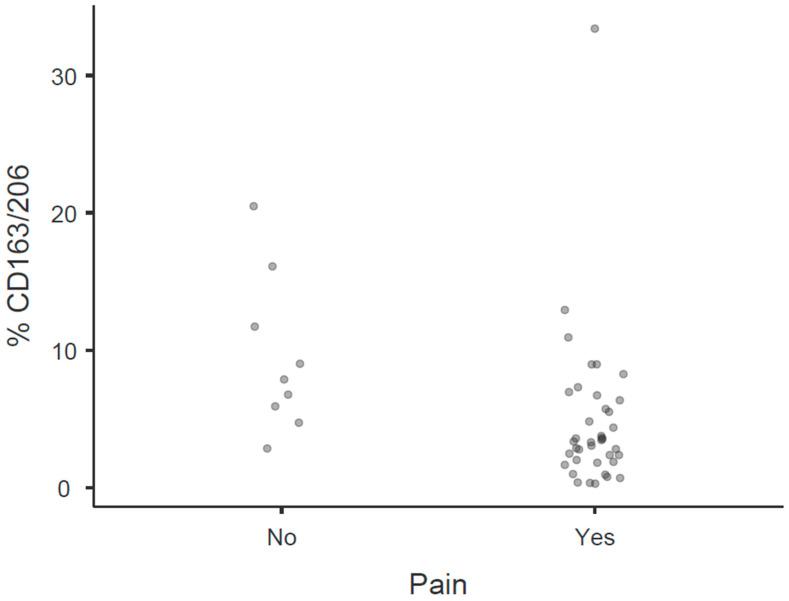
Main divergences in monocyte values and transmembrane proteins associated with the identification of pain. This figure consists of four scatter dot plots comparing “Yes” and “No” responses related to pain for four different variables: % CD206, % Lymphocytes, % CD163/CD206, and % Monocytes. Dot plot for % CD206. The “Yes” pain group is concentrated at lower percentage values, while the “No” pain group covers a broader range higher values. Dot plot for % Lymphocytes. The “No” pain group is more evenly distributed compared to the “Yes” pain group, which has a more focused distribution. Dot plot for % CD163/CD206. The “Yes” pain group again shows more concentration at lower values, with the “No” pain group spreading over a larger area. Dot plot for % Monocytes. Similar patterns are observed as the “Yes” pain group has a concentrated distribution, whereas the “No” pain group spans a wider range. Each plot includes a density curve to illustrate the distribution within each group. %—percentage; CD—cluster of differentiation.

In Figure 1, the main individual patient characteristics showing strong associations with pain are visualized. Similarly, Figure 2 displays the principal divergences in monocyte values and their transmembrane proteins were found to be associated with pain identification. Within this exploratory cohort, receiver operating characteristic (ROC) analysis indicated very high areas under the curve (AUCs), consistently above 0.900 (*p* < 0.001), suggesting strong discriminative potential for identifying cut-off points for these variables within our sample. However, it is important to note that these high AUC values are preliminary and reflect the findings within this specific study population, requiring external validation.

Table 3 presents the AUC values, cut-off points, sensitivity, specificity, positive predictive value (PPV), and negative predictive value (NPV) for age, GFR, and MNA observed in this study. As shown, we were able to identify exploratory cut-off points for patients with pain (e.g., age equal to or above 68 years, GFR equal to or lower than 90 mL/min/1.73 m^2^, and MNA equal to or under 12). These preliminary findings suggest that older patients, patients with kidney dysfunction, and those at risk of malnutrition or malnourished may be more likely to have pain in populations similar to our cohort. It is crucial to interpret these findings as exploratory, as the high-performance metrics are specific to the present study’s dataset and require validation in larger, independent studies.

After removing all variables that show multicollinearity [variance inflation factor (VIF) > 10] and the percentage of platelets expressing CD59, seven variables remain, namely age, dementia, percentage of lymphocytes, total monocytes, classic monocytes expressing CD163, intermediate monocytes, and non-classical monocytes.

**Figure 3:**
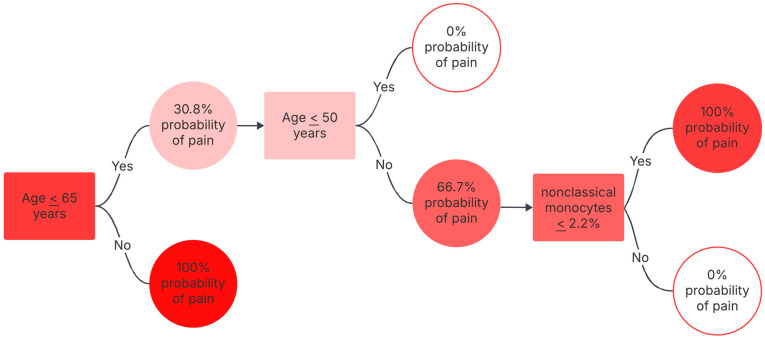
Classification tree algorithm of the presence of pain by age. All patients above 65 years had pain, and none of the patients with less than 50 years had pain. All patients aged between 50 and 65 that had a percentage of non-classic monocytes below 2.2% had pain. This figure shows the results of the classification tree, which converged identifying age and MonNC as pain discriminators. % = percentage; NCMon = non-classic monocytes.

The logistic regression model does not converge, resulting in overfitting as it accurately predicts all cases. The predictive regressive model was developed using regression analysis and dimension enhancement through forward feature selection. The classification tree algorithm, as depicted in Figure 3, provided an interpretable model for pain presence within our sample. This model indicated that, in this cohort, all patients over 65 years reported pain. Furthermore, among individuals under 50 years old, no pain cases were identified. For patients aged between 50 and 65 years, the tree suggested that those with a percentage of non-classic monocytes below 2.2% were associated with pain. Therefore, based on this classification tree derived from our limited dataset, all individuals over 50 years old were identified as having pain, with specific conditions for those between 50 and 65. These findings should be considered indicative of patterns within our study population and warrant further investigation in broader cohorts.

Given that we have few patients between 51 and 65 years old, these are preliminary results that need to be confirmed if possible, in larger samples.

All evaluated parameters appear to be independent of gender. Still, there is a statistically significant difference in age (*p* = 0.001), GFR (*p* = 0.001), percentage of classical monocytes expressing CD163 (*p* = 0.035), and percentage of non-classical monocytes (*p* = 0.025) between patients with and without dementia. Increasing the sample will be necessary to draw more robust conclusions in multivariate terms.

## 5. Discussion

This exploratory study aimed to identify potential pain biomarkers in patients managed by a specialized palliative care team. Through multivariate analysis, we examined personal characteristics, monocyte subsets, platelets, and selected transmembrane proteins in peripheral blood cells. Within our specific cohort, we found age, renal function, and nutritional status to be strongly associated with the presence of pain. We also explored the integration of these clinical parameters with potential pain biomarkers in peripheral blood cells (platelets and monocytes), previously identified in our univariate analyses [29,39]. These included the percentage of lymphocytes, total monocytes, classic monocytes expressing CD163, intermediate monocytes, and non-classical monocytes.

Platelets are increasingly recognized as active participants in inflammation and pain modulation, not merely as agents of hemostasis. The release of alpha-granule contents, including pronociceptive substances like serotonin, histamine, and bradykinin, is a key mechanism by which activated platelets contribute to nociception. CD62p (P-selectin) is a well-established marker of platelet activation and degranulation, mediating platelet–leukocyte interactions that drive inflammatory responses [24,25,26,27,28]. Although CD62p did not emerge as a significant independent predictor in our multivariate analysis, its mechanistic link to pain via the release of these mediators underscores the broader involvement of platelet activation in pain pathways.

Our finding of significantly lower CD59 expression on platelets in patients with pain (as shown in Table 1) offers a compelling mechanistic insight. CD59 is a crucial complement regulatory protein, specifically inhibiting the formation of the Membrane Attack Complex (MAC) on cell surfaces. A reduction in platelet surface CD59 could render these cells more vulnerable to complement-mediated activation or injury, or it could be a consequence of sustained platelet activation [29]. Mechanistically, heightened complement activation on platelets, or increased platelet vulnerability, could lead to direct platelet activation, the release of pro-inflammatory cytokines and chemokines, and increased platelet–endothelial interactions. These processes can amplify local inflammatory responses, contribute to endothelial dysfunction, and directly or indirectly enhance nociceptive signaling, thus exacerbating or perpetuating chronic pain [24,25].

These findings on platelet markers, alongside our observations in monocyte subsets, indicate a perturbation in immune cell function that may contribute to the pathophysiology of chronic pain in palliative care.

Older adults have more pain [48]. Besides a decline in global functionality and organ reserve, they also have a higher prevalence of diseases and comorbidities that cause pain [48,49]. Regarding dementia, pain can often be missed in people with these conditions [6,7]. Other studies showed that between 50% and 80% of patients with moderate to severe dementia experience pain daily [6,50]. Moreover, in our specific cohort, all patients with dementia and all patients above 65 years were observed to have chronic pain. This strong association observed within our dataset, as further highlighted by the classification tree, reinforces the significant challenge of pain in these vulnerable demographics and warrants further investigation.

Previous studies in older adults with chronic musculoskeletal pain have reported a strong relationship between pain and abnormal nutritional status [51]. High-calorie and unhealthy food leading to obesity can lead to many chronic pain conditions, especially spine- or joint-related pain [52]. On the other hand, evidence suggests that poor nutrition, such as malnutrition, unhealthy dietary behaviors, and poor and inadequate dietary intake, can play a significant role in the occurrence, prognosis, and maintenance of chronic non-cancer pain [53]. In addition, pain, age, and underweight were associated with a higher risk of malnutrition [51].

In our study, we used MNA scale to evaluate the nutritional status of our patients. This scale uses several parameters, such as daily food intake, weight loss, mobility, psychological stress or acute disease, BMI, and calf circumference. According to this scale, values between 24 and 30 points refer to normal nutritional status, values of 17 to 23 points report a risk of malnutrition, and below 17 points, the patient is considered to be malnourished [44,54]. In our study, all patients who did not suffer from pain had MNA values of 29 and 30 points (had normal nutrition), and most patients who suffered from chronic pain had MNA values equal to or below 20 points, indicating that they are at risk of malnutrition or malnourished.

Elma et al. (2022) described the underlying potential mechanisms that explain the interaction between malnutrition and chronic pain [53], namely the oxidative stress induced by diet, microbiota–gut–brain axis dysregulation, and carbohydrate intake is related to neuroinflammation and central sensitization. A decrease in adipose tissue may also improve pain sensitivity in chronic pain populations [53] and could also induce epigenetic changes, and alterations in gene expression in spinal cord and cerebral cortex are also related to chronic pain development [55]. On the other hand, pain decreases pleasure related to eating which may increase the risk of malnutrition [53].

Our study also suggested that patients with lower GFR, an indicator of renal dysfunction and overall health decline, tended to have more chronic pain. Other studies had similar results. Lambourg et al. (2021) reported a prevalence of 48% for chronic pain and 10% for neuropathic pain in patients with chronic kidney disease (CKD) [56]. Other studies reported prevalences of chronic severe pain in CKD patients up to 82% [57]. Given the diverse causes of pain in this population, it is not surprising that pain in CKD patients is often multifactorial (underlying systemic diseases, frailty, and painful syndromes such as ischemic limbs and neuropathies persist) [58].

The multi-analysis performed in this study, using the significative cellular and molecular biomarkers previously identified independently in the peripheral blood cells in our previous work [38,47], suggested the percentage of total monocytes and the percentage of classical monocytes are candidate predictive biomarkers in peripheral blood for the presence of pain. Regarding this subject, there is no consensus in the literature, as studies on patients with pain report different changes in the counts of monocyte populations and its subpopulations [31,33,36,39,59,60].

Austermann et al. (2022) referred that maladaptive innate monocytes and cytokines function contributes to chronic inflammation and pain [36]. Taylor et al. (2015) reported that pain and stress symptoms were significantly correlated to reduced percentages of classical and intermediate monocyte populations [61]. Sim et al. (2023) also reported that higher human monocyte levels are related to higher IL-10 and faster pain resolution [62]. Zhao et al. (2023) reported that non-classical macrophages are critical players in multiple endogenous pathways, promoting pain relief [37].

On the other hand, Loukov et al. (2018) reported that monocytes contribute to synovitis and disease pathogenesis in osteoarthritis, causing more pain [60]. Fotio et al. (2024) stated that circulating monocytes participates in pain chronification [38].

A notable finding in our study was the reduced percentage of intermediate monocytes in the pain group, which contrasts with reports of elevated intermediate monocyte subsets in several acute inflammatory disorders or active chronic diseases [31,32,34]. This apparent discrepancy may be attributed to the unique characteristics of our study population. Palliative care patients, especially older individuals with chronic non-oncological conditions and multiple comorbidities (including advanced dementia, malnutrition, and renal impairment), often experience a state of chronic, low-grade inflammation and immune dysregulation rather than an acute inflammatory flare. In such prolonged, debilitating conditions, persistent immune activation might lead to exhaustion, altered trafficking, or a phenotypic shift in intermediate monocytes, potentially resulting in their reduced representation in the peripheral circulation. Furthermore, the specific pathophysiology of non-oncological chronic pain in this frail, elderly population may involve distinct immune mechanisms that differ from those in other inflammatory diseases, warranting further investigation into these context-specific immune profiles.

Our multivariate analysis also suggests that the more relevant transmembrane proteins, particularly the percentage of monocytes expressing CD206 and the percentage ratio of CD163/CD206, in our results are in line with other studies, but in disagreement with others. We observed that patients with pain have a significant reduction in CD206 expression and there is a significant decrease in the CD163/CD206 ratio. Garrity et al. (2023) reported that intrathecal depletion of CD206 macrophages promoted sustained pain in mice [47]. Lin et al. (2025) reported that CD206 is associated with inflammatory pain resolution [63]. On the other hand, Silva et al. (2022) reported that higher number of macrophages and CD206 macrophage population accumulate at the nerve injury site and are associated with pain [40]. Wright et al. (2021) also reported that CD206 macrophages expressed markers consistent with a mature phenotype, with high levels of CD68 and CD163, as well as higher transcription of interleukin-10 (IL-10), being associated with pain in inflammatory bowel disease [41].

Nielsen et al. (2020) [42] reported that CD163 expression is increased in response to IL-10 stimulation (and it is associated with inflammation and pain), while IL-4 and IL-13 upregulate CD206 expression. It is associated with downregulation of inflammation and with pain control [42].

In our study, we observed a statistically significant decrease in the percentage of linfocytes and in intermediate and non-classic monocytes and an increase in the percentage of total monocytes. Furthermore, a decrease in the percentage of total monocytes expressing 206 and in the ratio of CD163/206 and of CD163 in classic monocytes were observed in patients with pain.

## 6. Limitations and Future Perspectives

This study, while providing valuable preliminary insights, is subject to several important limitations that warrant careful consideration.

Firstly, as a pilot and exploratory cross-sectional investigation, the relatively small sample size (n = 53) is a primary constraint. This limited sample size not only restricted the formation of evenly balanced groups but also impacted the robustness of our statistical analyses. Specifically, the non-convergence of the logistic regression model, likely due to challenges with multicollinearity and the number of variables relative to observations, necessitated the use of a classification tree algorithm. While the classification tree proved useful for identifying strong predictive patterns within our specific dataset, its results, including the high discriminative performance metrics (e.g., AUCs approaching 1.0) for individual variables, must be interpreted as exploratory. These high values are prone to optimism bias inherent in small-sample exploratory studies and may not generalize without rigorous external validation.

Secondly, the patient selection process involved the exclusion of a significant proportion of individuals (42 out of 95 initially screened) due to fragile conditions or end-of-life status. While these exclusions were ethically driven to ensure patient safety and comfort during blood collection, they introduce a potential selection bias. It is highly plausible that the excluded patients, being more severely ill, might exhibit different pain profiles and biomarker expressions. Consequently, our findings may not be fully generalizable to the most acutely frail or end-of-life palliative care populations, and are therefore more applicable to a less acutely fragile cohort.

Thirdly, the multifaceted nature of pain in patients with severe dementia, originating from various causes and mechanisms, along with heterogeneous responses to treatment, contributes to data variability. This study did not extensively explore additional confounding factors such as the precise duration of pain, specific prior or concurrent analgesic treatments received, or detailed medication histories, which could influence both pain assessment and biomarker profiles. Furthermore, the concomitant use of various medications, including analgesics (e.g., opioids), anti-inflammatory drugs (e.g., corticosteroids), and others (antidepressants, antiplatelets), represents a significant limitation. These drugs can directly modulate immune cell function and inflammatory pathways, as well as pain perception, thereby potentially confounding the observed associations between biomarkers and pain. While we have provided the usage rates in Supplementary Table S1, disentangling the independent effects of pain from those of medication on biomarker profiles is challenging in this cross-sectional study.

A further notable limitation is the amount of missing data for platelet CD59 (available for forty-six out of fifty-three patients, meaning seven patients had missing values), which led to its exclusion from multivariate analyses. This missingness (approximately 13% of the cohort) was primarily systematic, likely influenced by challenges in obtaining high-quality samples from a fragile palliative care population and technical limitations during processing for this specific marker. While available univariate data for CD59 are presented in Supplementary Table S1, its omission from key multivariate models weakens the mechanistic interpretation of its potential role as a biomarker, as its interactions with other predictors could not be fully assessed due to the methodological concerns regarding systematic missingness.

Moving forward, a confirmatory, multi-center study with a significantly larger and more diverse cohort of palliative care patients is essential to validate these preliminary findings. Such a study would enable more robust statistical modeling (potentially resolving logistic regression convergence issues), allow for the independent validation of the candidate biomarkers identified here, and help to establish their generalizability. Future research should also delve into the dynamic changes in these biomarkers over time, their response to analgesic interventions, and a more comprehensive analysis of clinical covariates, including careful consideration and control for confounding medication effects, such as opioid dose, type, and duration, on both pain assessment and immune profiles, to build more sophisticated and reliable predictive models for pain detection and management in this vulnerable patient population.

## 7. Conclusions

This exploratory study provides preliminary evidence, suggesting that certain individual characteristics, including renal impairment, malnutrition, and advanced age, are associated with pain in palliative care patients. Furthermore, we identified several potential biological biomarkers, such as changes in lymphocyte percentage (significant decrease in patients with pain), total monocyte percentage (significant increase in patients with pain), non-classical monocyte percentage (strong association with pain in patients aged between 51 and 65 years), and alterations in monocyte CD206 expression and the CD163/CD206 ratio (significant reductions in patients with pain).

Building upon our previous work, this pilot investigation represents an initial integrative effort to combine specific platelet and monocyte biomarkers with clinical patient characteristics in palliative patients with non-oncological pain. Our findings underscore the potential utility of monocytes and their membrane proteins, alongside individual clinical parameters like renal functionality and nutritional status, as candidate pain biomarkers. These preliminary insights suggest avenues for earlier and more accurate identification and characterization of pain in this vulnerable patient population, warranting further validation in larger, definitive studies.

## Figures and Tables

**Table 2:** Characterization of pain, degree of functionality, general performance, and nutritional status of the 44 patients with pain.

Assessment	Average	SD	Min	Max	Percentiles		
					25th	50th	75th
**PAINAD (38 patients)**	5.42	1.55	2	8	5	5	6.5
**NS (6 patients)**	5.83	2.40	2	8	3.5	6.5	8.0
**PPS**	32.75	13.4	10	60	20	40	40
**Barthel**	5.34	1.51	3	7	4	6	7
**MNA**	12.41	7.397	3	30	5	10	19.3

n = number; SD = standard deviation; min = minimum; max = maximum; PAINAD = Pain Assessment in Advanced Dementia Scale; NS = Numeric Scale; PPS = Palliative Performance Scale; MNA = Mini Nutritional Assessment.

**Table 3:** Values of AUC, sensitivity, specificity, positive predictive value, and negative predictive value related to age, GFR, and MNA.

Assessment	AUC	Cut-Off Point	Sensitivity	Specificity	PPV	NPV
Age	0.984	≥68	90.91%	100%	100%	69.23%
GFR	0.975	≤90	90.91%	100%	100%	69.23%
MNA	0.975	≤12	95.45%	100%	100%	81.82%

GFR = glomerular filtration rate; MNA = Mini Nutritional Assessment; AUC = Area Under the Curve; PPV = positive predictive value; NPV = negative predictive value; % = percentage.

## Data Availability

Dataset available on request from the corresponding author.

## Supplementary Materials

The following supporting information can be downloaded at: https://www.mdpi.com/article/10.3390/biomedicines14010176/s1, Table S1: Statistical analysis of monocytes, platelets and their specific receptors under study.
