# Supplementary material for: Peripheral Blood Cells and Clinical Profiles as Biomarkers for Pain Detection in Palliative Care Patients
Source: Biomedicines. 2026 Jan 14;14(1):176. doi: 10.3390/biomedicines14010176 (PMC12839203; doi:10.3390/biomedicines14010176)
Supplement: Supplementary file 1 [file biomedicines-14-00176-s001.zip › biomedicines-4018419-supplementary.pdf]

Table S1- Statistical analysis of monocytes, platelets and their specific receptors under study

	Dor	N	Média	Desvio-padrão	Mínimo	Máximo	Percentis 25th	Percentis 50th	Percentis 75th	p
Granulócitos	Não	9	63,68	13,80	46,10	85,50	50,80	66,00	69,80	0,080
	Sim	39	70,89	10,18	54,10	91,00	63,10	70,00	77,90	
Linfócitos	Não	9	29,06	13,22	7,30	45,90	23,10	26,40	41,40	0,042
	Sim	39	21,29	9,24	4,10	38,50	15,45	22,20	29,25	
Monócitos totais	Não	9	4,19	1,45	2,40	6,90	3,20	4,20	4,50	0,045
	Sim	39	5,71	2,09	1,40	10,80	4,40	5,60	6,90	
% pos 11c	Não	9	88,52	7,23	77,20	98,40	85,10	91,20	92,30	0,995
	Sim	39	88,48	16,13	16,80	99,20	87,95	95,40	97,10	
MIF 11c	Não	9	816,98	313,70	578,30	1610,00	630,80	742,30	816,20	0,361
	Sim	39	857,87	260,91	514,60	1515,20	667,80	852,70	987,90	
% pos 86	Não	9	32,69	12,27	18,10	59,70	26,40	27,80	36,70	0,081
	Sim	39	23,93	13,49	1,40	59,80	14,30	23,00	31,80	
MIF 86	Não	9	634,20	20,58	613,10	666,10	617,40	633,30	644,10	0,701
	Sim	39	639,47	39,43	590,90	781,30	611,85	631,30	665,65	
% pos 163	Não	9	85,63	6,36	73,60	91,90	81,30	88,70	90,10	0,342
	Sim	39	82,07	9,46	59,20	93,90	74,90	84,10	89,45	
MIF 163	Não	9	3061,99	1033,45	1310,60	4061,40	2567,20	3423,00	3858,60	0,375
	Sim	39	2500,81	1803,97	1122,40	11930,70	1591,95	1918,60	2970,80	
%pos 206	Não	9	14,08	7,80	3,40	23,40	6,80	15,40	21,30	0,017
	Sim	39	8,01	6,31	1,10	33,60	3,70	6,50	9,70	
MIF 206	Não	9	534,29	54,31	463,10	621,70	486,90	542,50	556,90	0,237
	Sim	39	493,06	99,28	382,60	989,50	429,60	471,40	509,80	
% 11c/86 +	Não	9	31,76	12,10	17,60	59,60	26,20	27,50	35,50	0,078

	Sim	39	23,24	12,89	1,30	59,80	14,20	22,50	30,60	
% 163/206+	Não	9	9,50	5,69	2,90	20,50	5,90	7,90	11,70	0,028
	Sim	39	4,79	5,58	0,30	33,40	1,95	3,40	6,05	
Monócitos clássicos	Não	9	85,27	5,39	77,90	96,00	83,50	84,50	88,20	0,003
	Sim	39	91,06	4,22	78,50	96,90	89,90	92,10	94,00	
% pos 11c (2)	Não	9	91,94	8,30	76,50	100,00	88,40	96,60	97,80	0,621
	Sim	39	93,64	9,40	62,40	100,00	92,75	97,30	99,10	
MIF 11c (2)	Não	9	740,59	286,19	519,10	1460,30	571,20	699,20	740,60	0,190
	Sim	39	825,35	260,68	483,80	1512,30	620,20	797,10	949,55	
% pos 86 (2)	Não	9	30,12	12,28	13,50	56,60	22,80	28,30	35,00	0,097
	Sim	39	21,70	13,68	1,10	60,00	11,40	20,10	29,70	
MIF 86 (2)	Não	9	592,10	13,74	579,50	624,20	581,70	590,50	596,10	0,792
	Sim	39	593,09	23,84	553,90	652,60	582,00	590,60	598,60	
% pos 163 (2)	Não	9	95,63	6,26	80,80	99,70	94,70	99,00	99,50	0,011
	Sim	39	87,99	9,36	67,30	99,70	79,55	90,20	94,50	
MIF 163 (2)	Não	9	3185,13	1119,81	1302,00	4252,70	2624,50	3687,80	4050,30	0,301
	Sim	39	2526,70	1799,54	1123,30	11783,00	1603,95	1941,30	3034,85	
%pos 206 (2)	Não	9	11,21	6,89	2,30	20,40	4,50	11,60	17,10	0,065
	Sim	39	6,71	6,33	0,80	35,40	3,00	5,20	8,00	
MIF 206 (2)	Não	9	503,38	48,62	447,80	580,30	462,80	485,80	541,10	0,332
	Sim	39	472,91	89,77	405,60	956,40	425,65	457,90	489,80	
% 11c/86 + (2)	Não	9	29,04	12,10	13,00	56,60	22,70	27,20	30,70	0,095
	Sim	39	20,95	12,98	1,00	60,00	11,35	20,00	28,25	

% 163/206+ (2)	Não	9	11,09	6,83	2,20	20,20	4,40	11,50	16,70	0,052
	Sim	39	6,52	6,06	0,80	34,10	2,95	5,10	7,85	
%Monõcitos intermédios	Não	9	5,71	2,65	1,60	9,30	3,70	6,20	6,90	0,008
	Sim	39	3,14	1,98	0,10	10,90	2,00	2,80	4,20	
% pos 11c (3)	Não	9	98,93	1,02	97,20	100,00	98,10	99,10	99,80	0,609
	Sim	39	98,38	3,14	83,60	100,00	98,60	99,60	99,80	
MIF 11c (3)	Não	9	1769,12	601,10	1052,20	2831,30	1257,40	1671,00	1922,30	0,903
	Sim	39	1743,82	551,78	790,60	3042,00	1353,30	1564,40	2128,00	
% pos 86 (3)	Não	9	77,98	9,49	68,00	93,30	70,30	72,40	84,20	0,225
	Sim	39	68,51	22,49	12,10	94,50	61,50	73,40	86,75	
MIF 86 (3)	Não	9	813,70	90,31	730,20	1011,00	748,10	779,90	845,40	0,293
	Sim	39	853,42	103,13	667,40	1079,00	780,10	851,60	920,55	
% pos 163 (3)	Não	9	88,12	9,58	66,30	99,20	85,00	91,50	93,20	0,066
	Sim	39	79,71	13,85	39,20	99,40	73,05	81,80	89,65	
MIF 163 (3)	Não	9	2397,87	1125,53	1097,40	5119,50	2137,10	2217,40	2340,00	0,181
	Sim	39	2843,90	3818,24	1096,30	23910,90	1487,20	1767,50	2144,65	
%pos 206 (3)	Não	9	43,71	17,61	18,20	73,40	30,50	40,20	55,80	0,183
	Sim	39	35,74	15,55	6,10	67,40	24,35	33,80	45,85	
MIF 206 (3)	Não	9	537,43	70,06	436,20	623,00	472,40	527,40	607,00	0,863
	Sim	39	548,42	185,49	66,30	1370,50	457,85	503,40	594,45	
% 11c/86 + (3)	Não	9	77,83	9,57	68,00	93,30	69,80	72,40	84,20	0,226
	Sim	39	68,41	22,45	12,10	94,50	61,50	72,00	86,75	

% 163/206+ (3)	Não	9	41,52	17,98	16,60	72,80	29,80	32,90	54,30	0,161
	Sim	39	33,19	15,33	5,40	66,10	22,20	29,90	45,15	
%Monócitos não clássicos	Não	9	5,53	3,26	1,30	9,60	3,80	4,80	8,60	0,007
	Sim	39	2,79	2,50	0,10	9,70	0,95	2,20	3,60	
% pos 11c (4)	Não	9	78,06	30,36	7,10	99,50	80,60	89,20	94,90	0,822
	Sim	39	78,71	26,20	13,80	99,40	72,00	91,00	97,75	
MIF 11c (4)	Não	9	1612,77	623,38	849,50	2798,80	1199,40	1591,70	1951,50	0,629
	Sim	39	1713,72	548,21	474,00	2770,50	1446,40	1727,10	2106,35	
% pos 86 (4)	Não	9	58,79	25,97	3,40	87,10	57,70	63,80	72,00	0,586
	Sim	39	64,22	26,89	1,80	95,40	53,75	74,10	83,70	
MIF 86 (4)	Não	9	790,99	82,09	722,40	957,10	727,30	757,30	823,90	0,132
	Sim	39	854,27	121,93	659,90	1271,30	778,25	823,40	931,80	
% pos 163 (4)	Não	9	14,61	12,39	2,60	43,30	7,30	11,50	18,60	0,538
	Sim	39	12,63	7,61	2,70	38,30	8,25	10,60	13,90	
MIF 163 (4)	Não	9	813,05	196,72	578,00	1242,10	717,50	825,50	839,50	0,532
	Sim	39	885,05	327,74	640,60	2098,30	723,85	770,20	851,45	
%pos 206 (4)	Não	9	17,04	16,27	2,30	57,50	8,10	13,70	15,70	0,191
	Sim	39	11,41	9,17	0,90	33,00	4,90	7,90	14,40	
MIF 206 (4)	Não	9	634,73	203,54	378,20	967,20	456,50	661,70	777,90	0,612
	Sim	39	572,90	347,49	322,10	1950,00	399,75	444,40	527,70	
% 11c/86 + (4)	Não	9	58,69	26,03	3,20	87,10	57,20	63,80	72,00	0,586
	Sim	39	64,11	26,90	1,80	95,40	53,60	74,10	83,60	

% 163/206+ (4)	Não	9	4,12	4,69	0,40	15,90	1,60	2,90	4,30	0,570
	Sim	39	3,81	4,89	0,00	24,40	1,15	2,40	3,45	
% pos CD36 / glicoproteína IV	Não	5	94,89	3,21	90,21	98,53	93,60	95,15	96,96	0,670
	Sim	41	92,56	11,96	55,96	99,84	91,19	98,38	99,25	
MIF CD36 (median intensity fluorescence - medida de quantidade)	Não	5	4615,39	992,35	3634,96	5751,40	3795,82	4316,84	5577,91	0,241
	Sim	41	5827,74	2237,95	2359,62	10058,95	4157,79	5694,57	7699,70	
% pos CD49f (molécula de adesão)	Não	5	98,81	0,41	98,25	99,29	98,61	98,82	99,10	0,511
	Sim	41	95,73	10,31	56,44	99,88	98,74	99,26	99,53	
MIF CD49f	Não	5	4484,02	1220,29	2838,28	6263,74	4217,64	4529,21	4571,23	0,605
	Sim	41	4732,71	982,74	2674,28	7040,98	4113,69	4689,75	5400,05	
% pos CD61 (ITG33 - integrina beta 3)	Não	5	98,78	0,52	98,13	99,45	98,52	98,69	99,12	0,518
	Sim	41	95,75	10,31	56,49	99,88	98,89	99,41	99,54	
MIF CD61	Não	5	13189,47	2327,06	9485,05	15285,32	12650,67	13574,08	14952,21	0,560
	Sim	41	14274,27	4026,27	3228,61	18842,82	13193,64	15876,83	16736,99	

% pos CD62P (p-selectina)	Não	5	18,85	20,50	3,24	50,23	4,28	7,33	29,19	0,916
	Sim	41	19,97	22,41	1,08	82,58	4,73	9,56	25,32	
MIF CD62P	Não	5	759,41	136,12	650,57	971,84	670,23	684,81	819,60	0,632
	Sim	41	1299,68	2478,98	426,03	16607,40	701,16	787,29	1032,19	
% pos CD59 (proteína inibidora da ativação do complemento)	Não	5	11,75	7,20	3,48	21,02	8,14	8,79	17,32	0,047
	Sim	41	2,72	4,10	0,06	20,68	0,55	1,37	3,03	
MIF CD59	Não	5	1463,17	443,02	1084,58	2010,15	1095,49	1256,64	1868,99	0,537
	Sim	41	1761,61	833,18	465,88	3842,40	1178,09	1424,30	2268,86	
% pos CD40 (receptor da familia do TNF)	Não	5	0,34	0,35	0,05	0,94	0,12	0,28	0,30	0,157
	Sim	41	0,07	0,09	0,01	0,44	0,02	0,04	0,06	
MIF CD40	Não	5	1369,01	428,92	1083,02	2096,73	1096,15	1150,76	1418,40	0,837
	Sim	41	1471,12	480,91	813,16	2891,16	1077,54	1303,92	1712,68	
